# The effect of prophylactic esketamine in labor and cesarian delivery on the prevention of postpartum depression (PPD): A systematic review and meta-analysis of randomized controlled trials

**DOI:** 10.1192/j.eurpsy.2024.252

**Published:** 2024-08-27

**Authors:** A. Kozhokar Mikhaylovskaya, A. Q. Shimit

**Affiliations:** ^1^Department of Medicine, Universitat Internacional de Catalunya, Sant Cugat del Vallès, Spain; ^2^Department of Medicine, Pontifical Catholic University of Poços de Caldas, Poços de Caldas, Brazil

## Abstract

**Introduction:**

Postpartum depression (PPD) is a common psychiatric illness affecting maternal health, which can lead to poor outcomes for the infant, mother and family. Since the usual pharmacological treatment has low efficacy and a delayed onset of action, new treatment options should be explored. A recent meta-analysis demonstrated positive effects of racemic ketamine on PPD, but limited evidence is available on its more potent derivative esketamine.

**Objectives:**

To determine the effect of esketamine administered prophylactically during labor on the risk of incidence of PPD at 1 week and 6 weeks after delivery.

**Methods:**

PubMed, Scopus and GoogleScholar databases were searched for randomized controlled trials that studied the efficacy of esketamine that screened for PPD using the Edinburgh Postpartum Depression Scale (EPDS). Risk ratio was used to determine the effect of incidence on PPD. Heterogeneity was examined with I2 statistics. A random-effects model was used, as per moderate heterogeneity (I2=59%, p-value<0.05).

**Results:**

We included 7 RCTs with 1287 patients, 635 having received esketamine (49.3%). Patient-controlled intravenous analgesia (PCIA) or single intravenous dose during the delivery or cesarian section were the main drug delivery methods. Follow-up ranged from 4 weeks to 6 months, and EPDS cut-off scores for depression risk differed between studies, from 9 to 13 points. Dosages varied from 0.2mg/kg to 0.5mg/kg for single-dose administration and 0.1mg/kg to 1.25mg/kg for PCIA. Incidence of PPD at one week (RR: 0.459 95%CI 0.217-0.970; p<0.05; figure 1A) and at 6 weeks (RR: 0.470 95%CI 0.273-0.810; p<0.01; figure 1B) was significantly less common in patients who received esketamine during or after labor. Risk of bias was low in 5 studies and moderate in 2 studies. Risk of publication bias is significant.

**Image:**

**Figure:**
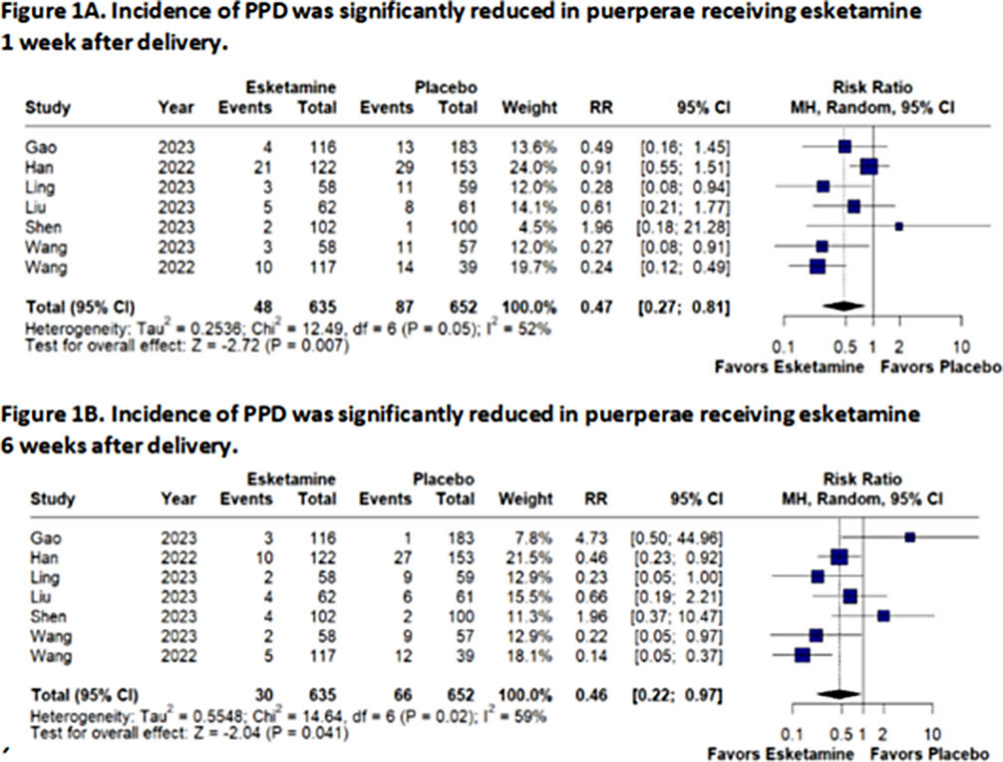

**Conclusions:**

Prophylactic esketamine seems to improve EPDS scores in women at one and six weeks after birth. A more thorough analysis of the adverse effects on maternal and neonatal health are required, and long-term benefits are not fully understood. Larger multicenter studies would be a welcome addition to the issue at hand.

**Disclosure of Interest:**

None Declared

